# A psychoeducational group intervention for family and friends of youth with borderline personality disorder features: protocol for a randomised controlled trial

**DOI:** 10.1186/s40479-018-0090-z

**Published:** 2018-07-25

**Authors:** Jennifer Betts, Jessie Pearce, Ben McKechnie, Louise McCutcheon, Sue M. Cotton, Martina Jovev, Victoria Rayner, Mirra Seigerman, Carol Hulbert, Catharine McNab, Andrew M. Chanen

**Affiliations:** 1 Orygen, The National Centre of Excellence in Youth Mental Health, 35 Poplar Rd, (Locked Bag 10), Parkville VIC, Melbourne, 3052 Australia; 2 Centre for Youth Mental Health, The University of Melbourne, Melbourne, Australia; 3 Orygen Youth Health, Northwestern Mental Health, Melbourne, Australia; 4 Melbourne School of Psychological Sciences, The University of Melbourne, Melbourne, Australia

**Keywords:** Borderline personality disorder, Early intervention, Randomised controlled trial, Treatment, Youth

## Abstract

**Background:**

Caring for a person with borderline personality disorder is associated with poor outcomes including elevated psychological distress and burden. This study will compare the effectiveness of two brief psychoeducational programs for carers of youth presenting for early intervention for borderline personality disorder features. The protocol for this study is presented here.

**Methods:**

The study is a single-centre parallel group, randomised controlled trial. As a family unit, relatives, partners and friends (‘carers’) are randomly allocated to one of two treatment arms to receive either an online borderline personality disorder psychoeducation program, or both the online psychoeducation group and a face-to-face group program, *Making Sense of Borderline Personality Disorder*. Carers are assessed at baseline and follow-up (4 weeks after the intervention). It is expected that participants who received the combined group and online programs will have better outcomes than those who received the online program alone. The primary outcome is carer burden, assessed using the negative appraisal subscales of the Experience of Caregiving Inventory. Secondary outcomes include positive experiences of caregiving, coping, self-rated personality disorder knowledge, psychological distress, expressed emotion and quality of life.

**Discussion:**

This will be the first published evaluation of a psychoeducational intervention for carers of youth with borderline personality disorder features using a randomised controlled trial design. The results have the potential to inform clinicians and carers about the effectiveness of brief interventions designed to support families and friends of young people with borderline personality disorder, and what medium those interventions should utilise.

**Trial registration:**

Prospectively registered with the Australian New Zealand Clinical Trial Registry ACTRN12616000304437 on 08 March 2016.

## Background

Borderline personality disorder (BPD) is a common and severe mental disorder comprising difficulties with affect regulation, impulsivity, interpersonal relationships and identity [1]. BPD usually has its onset during adolescence and early adulthood [2] and has been shown to be a reliable and valid diagnosis during these developmental periods [1, 3]. Families and friends (henceforth “carers”) of individuals with BPD experience high rates of psychological symptoms, including anxiety and depression [4, 5], objective and subjective burden, and grief [6, 7]. These rates are higher than the general population [4] and greater than carers of individuals with other severe mental illnesses [6, 7]. Such rates might arise from the experience of caring for a loved one with BPD and/or from difficulties (e.g., psychopathology) that might cluster within families of those with BPD.

Carers want information about BPD [8–10]. However, few psychoeducational interventions have been developed and, to the authors’ knowledge, just three of these programs have been subjected to formal empirical evaluation: ‘Dialectical Behaviour Therapy - Family Skills Training’ (DBT-FST; [11, 12]); ‘Family Connections’ [13] and ‘Staying Connected when Emotions Run High’ [14]. These evaluations indicate that psychoeducational programs consistently reduce carer burden and grief and increase perceived capacity to cope [13–15], but their effects upon psychological distress have been mixed [15, 16].

The above programs were primarily designed for carers of adults, although DBT-FST has been applied to a younger age group (13 to 18 years, [16]). Our team has developed and empirically tested the first psychoeducational group program designed specifically for carers of youth with BPD features. The *Making Sense of BPD* program (MS-BPD; [17]) accommodates the needs of carers of those with early-stage disorder and places BPD in an appropriate developmental context. In a pre-post, repeated measures design pilot study, MS-BPD was associated with reduced subjective burden (i.e., feelings and attitudes, such as shame) and increased personality disorder knowledge, but not with changes in objective burden (e.g., financial problems) or distress [17]. Several methodological issues limit the generalisability of these findings. The absence of a comparison group meant that changes could not solely be attributed to MS-BPD. Most participants completed the post-intervention measures on the day of the final MS-BPD session (day 15) and it is unclear what effect the program might have had on outcomes with longer follow-up. It was assumed that each round of the group was the same, not accounting for potential group clustering effects. Further, the study relied on a self-report screening measure for BPD, rather than a diagnostic assessment, and had a modest sample size of 23 carers. The pilot study’s promising findings warrant testing with a larger randomised controlled trial (RCT) that addresses these limitations. This paper describes the protocol for such an RCT.

This RCT aims to evaluate the effectiveness of an online BPD psychoeducation program (Online), compared with the online program delivered in conjunction with MS-BPD (Online+MS-BPD) for carers of youth with BPD. The trial’s hypotheses are that at the primary end-point of week seven, participants receiving Online+MS-BPD will have superior results on the primary (carer burden) and secondary (positive experience of caregiving, coping, personality disorder knowledge, distress, expressed emotion and quality of life) outcome measures, compared with participants receiving Online.

## Methods

### Study design

The study is a single-centre parallel group, single-blinded RCT. The study was developed in accordance with Good Clinical Practice (GCP) Guidelines and Standard Protocol Items; Recommendations for Interventional Trials (SPIRIT; [18]). The trial is being conducted by Orygen, The National Centre of Excellence in Youth Mental Health (Orygen). It was approved by the Melbourne Health Human Research Ethics Committee (HREC2014.105) and conducted in accordance with the Declaration of Helsinki. The trial was funded by a Melbourne Health Grant In Aid (GIA-013-2015) and was prospectively registered (ACTRN12616000304437). Members of Orygen’s Sponsor Operations Department monitors the trial.

### Study setting

The study is being conducted at the Helping Young People Early (HYPE) program at Orygen Youth Health (OYH) [19], a publically-funded youth mental health service in Melbourne, Australia. HYPE provides specialist early intervention for youth with severe personality disorder, offering clinical case management, individual cognitive analytic therapy (CAT) and general psychiatric care [19]. HYPE offers indicated prevention [20] and early intervention to young people with three or more DSM-5 BPD criteria, as there is evidence that ‘sub-threshold’ features of BPD are clinically significant [21]. There are no specific exclusions for HYPE, apart from meeting criteria for entry into Orygen’s first-episode psychosis program. The trial interventions are additional components of the care offered by the HYPE program to OYH clients with BPD features.

### Inclusion and exclusion criteria

Study participants are: (i) relatives, partners, or friends of a HYPE client; (ii) able to give informed consent; (iii) sufficiently fluent in English; and (iv) able to comply with study procedures. Carers are excluded if their client meets the trial’s exclusion criteria or if they have previously participated in MS-BPD or Online. Carers may not have a professional relationship with a client, for example, child protection workers or staff at residential care units are not eligible. The clients must be: (i) attending HYPE; (ii) aged 15 to 25 years (inclusive); (iii) able to give informed consent; (iv) sufficiently fluent in English; and (v) able to comply with study procedures. Clients are excluded if they are eligible for OYH’s first-episode psychosis program [22, 23].

### Discontinuation and withdrawal

Carers are discontinued or withdrawn if their participation interferes with appropriate clinical management of the client’s risk to self or others, consent is revoked, or an event (e.g. inappropriate behaviour in the MS-BPD group setting) leads to discontinuation at the discretion of the investigators. These participants remain in the sample to be analysed.

### Interventions

*MS-BPD* is a manualised group program designed for carers of young people with BPD features, informed by the principles of CAT. It consists of three two-hour sessions facilitated by two specialist youth mental health clinicians; the third session is co-facilitated by a family peer support worker, with lived experience of caring for a mentally-ill young person. MS-BPD is run in the evening over three consecutive weeks (i.e., on days one, eight and fifteen). It aims to provide an explanatory model of the causes of BPD, psychoeducation about the rationale for and nature of BPD treatment, along with information about common difficult relationship processes and possible ways to resolve these. The group is interactive, and participants are encouraged to ask questions and to share their experiences. Treatment completion is defined as attending two or more sessions.

The *Online* program was also developed specifically for carers of youth with BPD features. It comprises two modules: ‘Introduction to Early Intervention for Borderline Personality Disorder’ and ‘Caring For A Young Person with Borderline Personality Disorder - Information for Families and Friends’. The modules take approximately 30 and 20 min to complete, respectively, and include written material and video interviews with experienced clinicians, clients and parents. The program is self-directed, can be accessed multiple times, and is accessible at all times. Date, time and duration of each visit is recorded and treatment completion is defined as using the program for at least 80% of the mean time it takes to complete the entire program.

### Treatment integrity

MS-BPD integrity is maintained through regular facilitator supervision and use of the MS-BPD manual and standardised program resources (e.g., presentation slides). The fixed design of the Online program ensures its integrity.

### Outcome measures

The primary outcome is burden, defined as the combined total score of negative appraisal subscales of the Experiences of Caregiving Inventory (ECI; [24]). The secondary outcomes include positive experience of caregiving (ECI total positive appraisal subscale; [24]), coping (Coping Inventory for Stressful Situations; [25]), self-rated personality disorder knowledge (selected items from Personality Disorder Knowledge, Attitudes and Skills Questionnaire, modified for use with carers; [26]), distress (Kessler Psychological Distress Scale; [27]), expressed emotion (Family Questionnaire; [28]), and quality of life (Assessment of Quality of Life - 8 Dimensions and Quality of Life Enjoyment and Satisfaction Questionnaire - Short Form; [29, 30]). Subsidiary measures capture demographic, treatment/resource use and diagnostic information (e.g. Structured Clinical Interview for Diagnostic and Statistical Manual of Mental Disorders, Fourth Edition, Axis I and II disorders; [31, 32]). Table 1 lists the trial measures.

**Table 1:** Schedule of outcome measures

	Time point	
Measure	Baseline	Week Seven
*Primary Outcomes*		
Combined total negative appraisal subscales of the Experience of Caregiving Inventory (ECI)	✔	✔
*Secondary Outcomes*		
Combined total positive appraisal subscales of the ECI	✔	✔
Coping Inventory for Stressful Situations (CISS)	✔	✔
Personality Disorder Knowledge Attitudes and Skills Questionnaire (PDKASQ) ^a^	✔	✔
Kessler Psychological Distress Scale (K-10)	✔	✔
Family Questionnaire (FQ)	✔	✔
Assessment of Quality of Life (AQoL-8D)	✔	✔
Quality of Life Enjoyment and Satisfaction Questionnaire – Short Form (Q-LES-Q-SF)	✔	✔
*Subsidiary Measures*		
Demographics - carers	✔	✔
Carer-specific Resource Use	✔	✔
Demographics – clients ^b^	✔	
Treatment Data ^b^		✔
SCID-II Personality Questionnaire for BPD ^b^	✔	
Diagnosis DSM-IV SCID-I/P ^b^	✔	
BPD diagnosis DSM-IV SCID-II ^b^	✔	

aThree items, modified for use with carers rather than clinicians

b Measures completed with clients, not carers

*BPD* borderline personality disorder, *DSM-IV* Diagnostic and Statistical Manual, Fourth Edition, *SCID* Structured Clinical Interview for DSM-IV; SCID-I/P=SCID Axis I Disorders, Patient Version; SCID-II=SCID Axis II Disorders

### Procedure

**Fig. 1:**
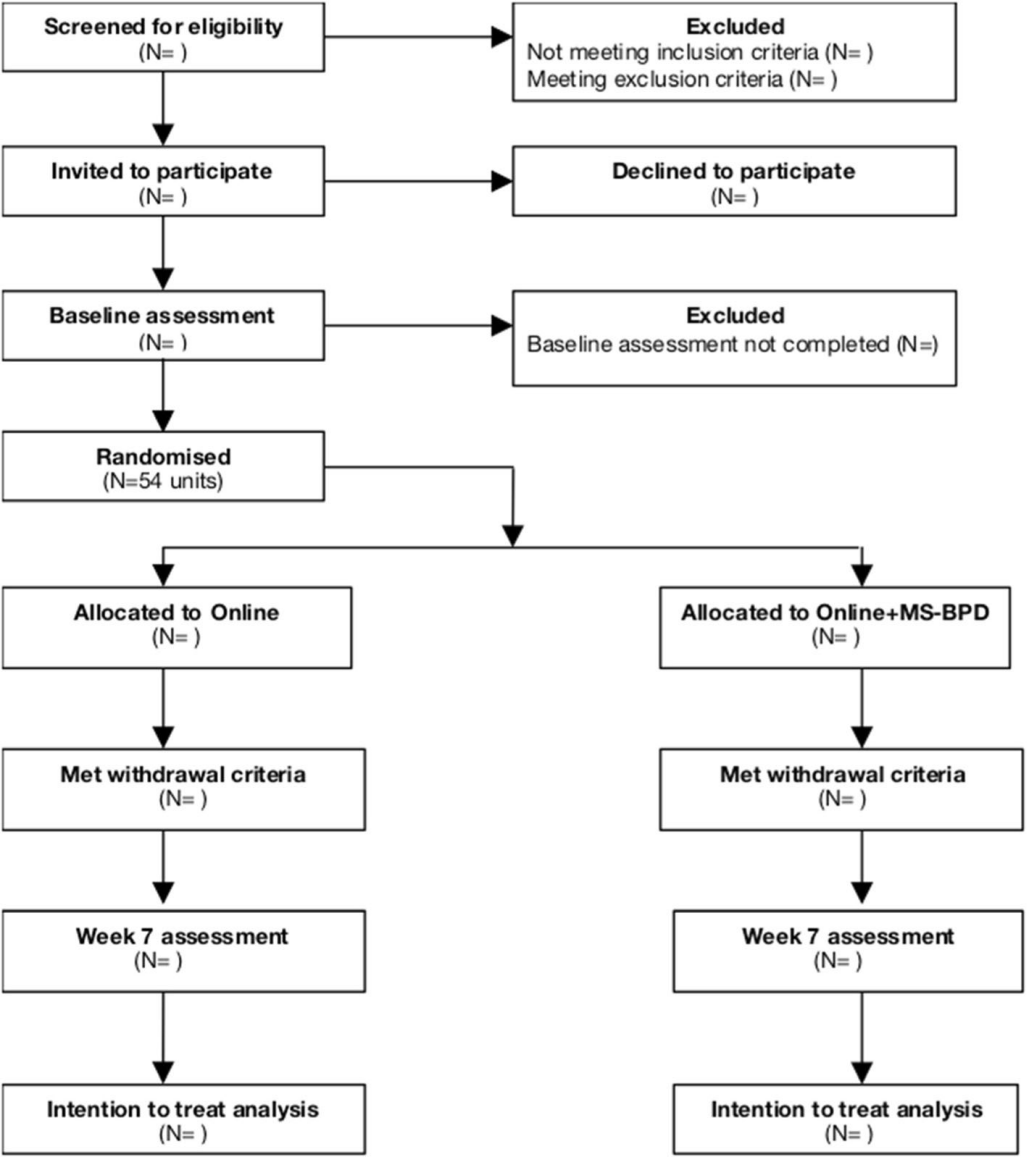
CONSORT flow diagram for MS-BPD trial

Participants (and a parent/legal guardian for minors) are asked to provide written informed consent. A carer’s participation is not dependent on their young person also consenting to participate. Carers who decline participation in the trial may still utilise the interventions. The interventions are delivered in rounds, approximately every 12 weeks. Information collected as part of routine clinical care [33] is extracted from consenting clients’ medical records. Carers are given baseline questionnaires up to 2 weeks prior to the forthcoming round, and upon completion of these, carers are randomly and consecutively assigned to the next Online or Online+MS-BPD as a family unit. Follow-up questionnaires are issued at week seven, (4 weeks after the intervention), and carers have up to 4 weeks to complete these. The baseline and follow-up questionnaires are self-rated, online or in hard copy. Figure 1 shows the participant flow chart.

### Randomisation and blinding

Randomisation occurs in a 1:1 ratio using a password-protected computer program with a sequence that was computer-generated by an independent statistician. Treatment allocation uses randomised permuted blocking, stratified by client’s sex and age (< 18 years old; 18 years is the mean age of HYPE clients). The trial statistician is blinded to group allocation, but due to the nature of the interventions and outcome measures (i.e., self-rated questionnaires), the participants, clinicians and researchers are not blinded.

### Data integrity

Most data are entered directly online by the participants. For data collected in hard copy, data entry verification will be undertaken on a random selection of 20 % of cases at each time point, with an a priori acceptable error rate of 0.5%.

### Statistical analysis

The analysis will be by intention-to-treat. Two sources of potential non-independence (clustering) of observations are: (1) participation in the intervention by more than one carer associated with an individual client (a ‘unit’); (2) the round of MS-BPD attended. Thus, the unit is considered a cluster and within the Online+MS-BPD arm, the MS-BPD round attended is considered the intervention cluster (IC). Such a design is referred to as a partially nested, clustered RCT [34]. To accommodate this design, mixed models will be used with unit and IC fitted as random factors [34]. Although low levels of missing data are expected, mixed models have the advantage of retaining the observations of participants who have missing data [35]. The models will be extended to include baseline and time varying covariates as well as categorical variables that might confound outcomes and require adjustment. A two-step approach will first identify individually significant and near-significant associations with outcome measures, and then simultaneously fit these variables in a multiple predictor model. This approach will be applied to each of the outcomes specified in the hypotheses. Adaptations of this approach will be used to explore predictors of the outcomes. Further, given that mothers have been the most frequent attendees of MS-BPD to date, exploratory analyses of this subgroup alone will occur. It is recognised that these analyses will have reduced power to detect change and the study has not been powered for subgroup analyses.

### Sample size determination

The power analysis must take into account potential clustering effects due to unit, as well as IC within the Online+MS-BPD arm [34]. The average number of participants per unit is 1.5 (*m*_*H*_). The responses of members from within the same unit might be more alike than responses between different units. It is difficult to estimate the magnitude of likely intraclass correlations but substantial effects must be considered, thus, the intraclass correlation coefficient was conservatively estimated to be at 0.2 (*ρ*_*εθ*_).

It is recognised that receiving the Online+MS-BPD intervention in a group setting might result in participants’ responses being more alike within each round than between rounds. This might arise due to effects associated with each round’s facilitators, along with events, experiences and relationships that emerge at each round. However, in the case of psychoeducation interventions, clustering effects are typically very low. This is due to the absence of any pre-existing clustering (in contrast to clustered RCTs), the programmatic nature of the intervention, and the limited amount of interaction participants have within the rounds. As such, we conservatively estimated the IC to be correlated at 0.03 (*ρ*_*θT*_). Our pilot data revealed that the average number of participants per MS-BPD round was 9.4 (*J*). Using these parameters, the maximum design effect would be 1.24.

Taking the above factors into account and assuming a correlation of 0.5 between baseline and outcome measures, a total sample of 54 units would have power of 80% to detect a difference of 0.5 standard deviations between the Online and Online+MS-BPD arms. This effect size is regarded as a medium size difference.

## Discussion

To the authors’ knowledge, this will be the first published RCT of any psychoeducational program for carers of youth with BPD. We have designed and previously empirically tested MS-BPD, the first psychoeducational group program for carers of youth with BPD features, that places BPD in an appropriate development context and addresses the needs of carers of youth, early in the course of the disorder [19]. By employing an RCT design and an ‘active’ comparison condition, this study will allow for a more rigorous test of the effectiveness of MS-BPD, compared with an online BPD program with a focus on psychoeducation.

The trial’s findings will help to clarify the extent to which psychoeducational programs for carers of youth with BPD features are effective, and to give feedback about their content. If the online program is found to be effective then it can be readily applied in clinical practice. The program only requires carers to have internet access, is of short duration, can be accessed multiple times, and can be completed in stages. These elements are likely to make the online program attractive to carers who are busy juggling a range of family, study and work commitments. It is also a low-intensity intervention for services to offer, a key issue in the utilisation of carer interventions [36]. If the trial finds that the online program is not effective, it might be that the design, content, or length of the program needs revision or that group processes are key in contributing to change.

If the combination of the online program and MS-BPD proves to be most effective, then this supports the implementation of more resource-intensive interventions, requiring a relatively greater investment by both carers (in attending an on-site group) and services (using specialist clinicians and family peer support workers in conducting the group). However, and importantly for uptake, specialist psychotherapy training (in CAT) is not required of those conducting the group. The manualised MS-BPD program, with its standardised materials, can easily be disseminated for use in a range of clinical settings. Factors that might be responsible for the combined Online+MS-BPD intervention being more effective might include: (i) validation of and connection with the experiences of other carers; (ii) shared problem solving; (iii) the opportunity to ask questions and to discuss or tailor the content to individual circumstances; and (iv) facilitators’ capacity to respond to dynamic group needs, depending upon how the material is understood or the feelings being expressed within the group. The specific effect of each factor or MS-BPD component has not been evaluated, but would be of interest for future research. If offering MS-BPD in addition to the online program does not improve outcomes over and above Online, then it might be that MS-BPD is more effective for carers with greater difficulties (e.g., higher levels of burden and distress), whereas the less intensive online program might be sufficient for carers experiencing fewer difficulties.

While this trial’s design attempted to overcome problems identified in previous studies, there are some anticipated limitations. This trial will not assess the maintenance of any changes over the medium- to long-term. Future studies would be strengthened by having additional follow-up assessments, longer than four to eight weeks post-intervention. Compared with participants in the Online arm, participants in the Online+MS-BPD arm might have greater exposure to psychoeducation, given that they will have the Online material repeated by MS-BPD facilitators. The trial results will therefore need to be interpreted with this in mind, with the Online usage data and MS-BPD attendance rates elucidating whether the ‘dose’ of psychoeducation differed between the trial’s arms. Further, reflective of ‘real-world’ clinical practice, carers are free to access additional carer-specific resources, which might confound the trial’s findings. OYH, for example, offers individual sessions with specialist family clinicians and peer support from trained family peer support workers. However, data on the use of such resources is being collected and will be controlled for in our analyses. Although beyond the scope of this trial, if the online program and MS-BPD prove to be effective for carers, an examination of whether improved carer outcomes might be associated with better outcomes for young people with BPD is warranted. To the authors’ knowledge, this relationship has not previously been empirically tested and will be an important extension of the field.

Notwithstanding these limitations, the trial design has several strengths. The trial benefits from broad inclusion criteria, with few exclusion criteria, which enables the recruitment of a sample that reflects the clients and carers who present to government-funded specialist mental health services in Melbourne, Australia. Carers representing a range of different types of relationships are included, rather than limiting the study to just parents, or mothers, as is commonly the case [7]. While much of the research to date has focused on those caring for females with BPD, this trial includes carers of both males and females with BPD [7]. BPD is assessed with a standardised clinical interview. Lastly, the manualised nature of MS-BPD and the pre-programmed MS-BPD and Online resources ensure treatment integrity.

In conclusion, this trial will test the effectiveness of an online psychoeducation intervention for carers of youth with BPD, alone or in combination with a face-to-face psychoeducation group. Results will have the potential to inform clinical decisions about which program, or combination of programs, to offer carers of youth with BPD features, as well as enabling carers to choose the style of intervention that best suits their needs.

## Abbreviations

ANZCTR: Australian New Zealand Clinical Trials Registry
BPD: Borderline personality disorder
CAT: Cognitive analytic therapy
DBT-FST: Dialectical Behaviour Therapy - Family Skills Training
ECI: Experiences of Caregiving Inventory
GCP: Good Clinical Practice
HREC: Human Research Ethics Committee
HYPE: Helping Young People Early program
IC: intervention cluster
MS-BPD: Making Sense of Borderline Personality Disorder program
OYH: Orygen Youth Health
RCT: Randomised controlled trial
SPIRIT: Guidelines and Standard Protocol Items; Recommendations for Interventional Trials

## References

[1] Leichsenring F, Leibing E, Kruse J, New AS, Leweke F (2011). Borderline personality disorder. Lancet.

[2] Chanen AM, McCutcheon L (2013). Prevention and early intervention for borderline personality disorder: current status and recent evidence. Br J Psychiatry Suppl.

[3] Chanen A, Sharp C, Hoffman P, Global Alliance for Prevention and Early Intervention for Borderline Personality Disorder. Prevention and early intervention for borderline personality disorder: a novel public health priority. World Psychiatry. 2017;16:215–6. 10.1002/wps.20429 PMC5428197 28498598

[4] Scheirs JGM, Bok S (2007). Psychological distress in caretakers or relatives of patients with borderline personality disorder. Int J Soc Psychiatry.

[5] Bailey RC, Grenyer BFS (2015). The relationship between expressed emotion and wellbeing for families and carers of a relative with Borderline Personality Disorder. Personal Ment Health.

[6] Bailey RC, Grenyer BFS (2013). Burden and support needs of carers of persons with borderline personality disorder: a systematic review. Harv Rev Psychiatry.

[7] Bailey RC, Grenyer BFS (2014). Supporting a person with personality disorder: a study of carer burden and well-being. J Pers Disord.

[8] Buteau E, Dawkins K, Hoffman P (2008). In their own words: Improving services and hopefulness for families dealing with BPD. Soc Work Ment Health..

[9] Dunne E, Rogers B (2013). “It’s Us That Have to Deal with it Seven Days a Week”: Carers and Borderline Personality Disorder. Community Ment Health J..

[10] Lohman MC, Whiteman KL, Yeomans FE, Cherico SA, Christ WR (2017). Qualitative Analysis of Resources and Barriers Related to Treatment of Borderline Personality Disorder in the United States. Psychiatr Serv..

[11] Fruzzetti AE, Santisteban DA, Hoffman PD, Dimeff LA, Koerner K (2007). Dialectical behavior therapy with families. Dialectical Behavior Therapy in clinical practice Applications across disorders and settings.

[12] Hoffman PD, Fruzzetti A, Swenson C (1999). Dialectical Behavior Therapy-Family Skills Training. Fam Process..

[13] Hoffman PD, Fruzzetti AE, Buteau E, Neiditch ER, Penney D, Bruce ML (2005). Family connections: a program for relatives of persons with borderline personality disorder. Fam Process..

[14] Bailey R. Caring for a person with personality disorder: A study of carer burden, support needs and interventions. University of Wollongong; 2014. http://ro.uow.edu.au/theses/4188/. Accessed 16 Mar 2017.

[15] Hoffman PD, Fruzzetti AE, Buteau E (2007). Understanding and engaging families: An education, skills and support program for relatives impacted by borderline personality disorder. J Ment Health..

[16] Ekdahl S, Idvall E, Perseius K-I (2014). Family skills training in dialectical behaviour therapy: the experience of the significant others. Arch Psychiatr Nurs..

[17] Pearce J, Jovev M, Hulbert C, McKechnie B, McCutcheon L, Betts J (2017). Evaluation of a psychoeducational group intervention for family and friends of youth with borderline personality disorder. Borderline Personal Disord Emot Dysregul..

[18] Chan A-W, Tetzlaff JM, Gøtzsche PC, Altman DG, Mann H, Berlin JA (2013). SPIRIT 2013 explanation and elaboration: guidance for protocols of clinical trials. BMJ..

[19] Chanen AM, McCutcheon L, Kerr IB, Sharp C, Tackett JL (2014). HYPE: A Cognitive Analytic Therapy-Based Prevention and Early Intervention Programme for Borderline Personality Disorder. Handbook of Borderline Personality Disorder in Children and Adolescents.

[20] Mrazek PB, Haggerty RJ (1994). Reducing Risks for Mental Disorders: Frontiers for Preventive Intervention Research. National Academy of Sciences.

[21] Thompson K, Jackson H, Cavelti M, Betts J, McCutcheon L, Jovev M, Chanen A. The clinical significance of subthreshold borderline personality disorder features in outpatient youth. J Personal Disord. In press. 10.1521/pedi_2018_32_330 30036169

[22] McGorry PD, Edwards J, Mihalopoulos C, Harrigan SM, Jackson HJ (1996). EPPIC: an evolving system of early detection and optimal management. Schizophr Bull..

[23] Cotton SM, Filia KM, Ratheesh A, Pennell K, Goldstone S, McGorry PD (2016). Early psychosis research at Orygen, The National Centre of Excellence in Youth Mental Health. Soc Psychiatry Psychiatr Epidemiol..

[24] Szmukler GI, Burgess P, Herrman H, Benson A, Colusa S, Bloch S (1996). Caring for relatives with serious mental illness: the development of the Experience of Caregiving Inventory. Soc Psychiatry Psychiatr Epidemiol..

[25] Endler NS, Parker JDA (1994). Assessment of multidimensional coping: Task, emotion. and avoidance strategies. Psychol Assess..

[26] Davies J, Sampson M, Beesley F, Smith D, Baldwin V (2014). An evaluation of Knowledge and Understanding Framework personality disorder awareness training: Can a co-production model be effective in a local NHS mental health Trust?. Personal Ment Health..

[27] Kessler RC, Andrews G, Colpe LJ, Hiripi E, Mroczek DK, Normand SLT (2002). Short screening scales to monitor population prevalences and trends in non-specific psychological distress. Psychol Med..

[28] Wiedemann G, Rayki O, Feinstein E, Hahlweg K (2002). The Family Questionnaire: development and validation of a new self-report scale for assessing expressed emotion. Psychiatry Res..

[29] Richardson J, Iezzi A, Khan MA, Maxwell A (2014). Validity and reliability of the Assessment of Quality of Life (AQoL)-8D multi-attribute utility instrument. Patient..

[30] Endicott J, Nee J, Harrison W, Blumenthal R. Quality of Life Enjoyment and Satisfaction Questionnaire. PsycTESTS Dataset. 1993; 10.1037/t49981-000. 8290681

[31] First MB, Spitzer RL, Gibbon M, Williams JBW (1995). Others. Structured clinical interview for DSM-IV axis I disorders.

[32] First MB. User’s Guide for the Structured Clinical Interview for DSM-IV Axis II Personality Disorders: SCID-II: American Psychiatric Press; 1997.

[33] Chanen AM, McCutcheon LK, Germano D, Nistico H, Jackson HJ, McGorry PD (2009). The HYPE Clinic: an early intervention service for borderline personality disorder. J Psychiatr Pract..

[34] Lohr S, Schochet PZ, Sanders E. Partially Nested Randomized Controlled Trials in Education Research: A Guide to Design and Analysis. NCER 2014-2000. National Center for Education Research. 2014. https://eric.ed.gov/?q=ED545532&id=ED545532.

[35] Hamer RM, Simpson PM (2009). Last observation carried forward versus mixed models in the analysis of psychiatric clinical trials. Am J Psychiatry..

[36] Eassom E, Giacco D, Dirik A, Priebe S (2014). Implementing family involvement in the treatment of patients with psychosis: a systematic review of facilitating and hindering factors. BMJ Open..

